# A Fluorinated Analogue of Marine Bisindole Alkaloid 2,2-Bis(6-bromo-1*H*-indol-3-yl)ethanamine as Potential Anti-Biofilm Agent and Antibiotic Adjuvant Against *Staphylococcus aureus*

**DOI:** 10.3390/ph13090210

**Published:** 2020-08-26

**Authors:** Raffaella Campana, Gianmarco Mangiaterra, Mattia Tiboni, Emanuela Frangipani, Francesca Biavasco, Simone Lucarini, Barbara Citterio

**Affiliations:** 1 Department of Biomolecular Sciences, University of Urbino Carlo Bo, 61029 Urbino, Italy; raffaella.campana@uniurb.it (R.C.); mattia.tiboni@uniurb.it (M.T.); emanuela.frangipani@uniurb.it (E.F.); 2 Department of Life and Environmental Sciences, Polytechnic University of Marche, 60131 Ancona, Italy; g.mangiaterra@pm.univpm.it (G.M.); f.biavasco@staff.univpm.it (F.B.)

**Keywords:** MRSA, marine bisindole alkaloids, antibiofilm activity, adjuvants agents, VBNC cells

## Abstract

Methicillin resistant *Staphylococcus aureus* (MRSA) infections represent a major global healthcare problem. Therapeutic options are often limited by the ability of MRSA strains to grow as biofilms on medical devices, where antibiotic persistence and resistance is positively selected, leading to recurrent and chronic implant-associated infections. One strategy to circumvent these problems is the co-administration of adjuvants, which may prolong the efficacy of antibiotic treatments, by broadening their spectrum and lowering the required dosage. The marine bisindole alkaloid 2,2-bis(6-bromo-1*H*-indol-3-yl)ethanamine (**1**) and its fluorinated analogue (**2**) were tested for their potential use as antibiotic adjuvants and antibiofilm agents against *S. aureus* CH 10850 (MRSA) and *S. aureus* ATCC 29213 (MSSA). Both compounds showed antimicrobial activity and bisindole **2** enabled 256-fold reduction (ΣFICs = 0.5) in the minimum inhibitory concentration (MIC) of oxacillin for the clinical MRSA strain. In addition, these molecules inhibited biofilm formation of *S. aureus* strains, and compound **2** showed greater eradicating activity on preformed biofilm compared to **1**. None of the tested molecules exerted a viable but non-culturable cells (VBNC) inducing effect at their MIC values. Moreover, both compounds exhibited no hemolytic activity and a good stability in plasma, indicating a non-toxic profile, hence, in particular compound **2**, a potential for in vivo applications to restore antibiotic treatment against MRSA infections.

## 1. Introduction

The diffusion of methicillin resistant *Staphylococcus aureus* (MRSA) strains is considered among the most common causes of health care-associated infections (HAIs) in hospitalized people all around the world [1]. Treatment of MRSA infections is limited, mainly because these microorganisms can develop resistance to multiple antibiotics [2,3]. Moreover, MRSA infections can be worsened by the ability of MRSA strains to grow as matrix-enclosed communities called biofilms, which promote adhesion and favor long-term survival on both biotic and abiotic surfaces [4,5].

Another strategy that MRSA have developed to resist antibiotic treatment is the ability to enter a state of dormancy, either as viable but non-culturable cells (VBNC) or as antibiotic persisters (APs) [6,7]. The complex architecture of biofilms, presenting different stressful microenvironments, may lead to a spatial-physiologic heterogeneity of the embedded bacterial population [8,9] and, in this peculiar survival structure, both VBNC cells and AP can be present. Considering that VBNC cells have been shown to tolerate a wide variety of stressors, including starvation, growth inhibiting temperatures, suboptimal salinity, suboptimal pH and antibiotics [10], their role in biofilm biology is of great concern in view of developing new therapeutic approaches. One of these includes the use of antibiotic adjuvants (i.e., molecules that have the potential to improve the effectiveness of an antibiotic against which bacteria have developed resistance), by reducing the bacterial resistance to it, hence prolonging the lifespan of life-saving drugs [11].

In this context, marine sessile organisms can represent a suitable source of bioactive products that are normally used to contrast bacteria diffused in the surrounding water [12]. These molecules have shown activity and selectivity against a wide spectrum of pharmacological targets, and their structures are often used as leads in drug discovery and development [13]. Among the various structural classes, bisindole alkaloids have attracted the attention of many researchers for their biological activities [14,15,16], especially their antimicrobial and antibiofilm activities [17,18,19]. Most of them present bromine and/or chlorine substitutions but there are no known examples of natural marine alkaloids containing fluorine. One of the possible reasons of the absence of fluorometabolites could be due to the very low abundance of fluoride ion (1.3 ppm) in the oceans with respect to chloride (Cl^−^ = 20,000 ppm) and bromide (Br^−^ = 70 ppm). On the other hand, about 20–25% of drugs on the market contain at least one fluorine atom. The very special effects of fluorine are very difficult to fully rationalize and its high presence in anthropogenic bioactive molecules has generally arisen from intense structure–activity relationship studies [20]. However, some of the effects of fluorine substitution are relatively straightforward to interpret such as the ability of fluorinated molecules to suppress metabolism relative to their hydrocarbon analogues. Moreover, organo-fluorine compounds are biologically and chemically more stable than the corresponding chlorine and bromine containing compounds [21].

**Figure 1:**
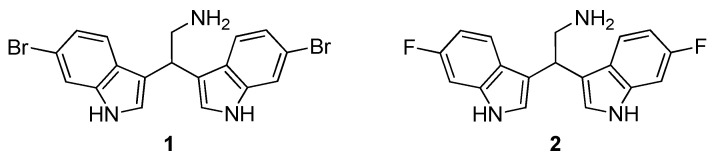
Marine bisindole alkaloid 2-bis(6-bromo-3-indolyl) ethylamine **1** and its fluorinated analogue **2**.

As a part of our ongoing investigations of the biological activities and possible applications of the marine bisindole alkaloid 2,2-bis(6-bromo-1*H*-indol-3-yl)ethanamine **1** [14,15,16,17,18] and for all the above described reasons, in the present work we focus our studies on natural compound **1** and its fluorinated analogue **2** [18] (Figure 1) to determine their potential as antibiotic adjuvants and antibiofilm agents against methicillin-susceptible (MSSA) and methicillin-resistant (MRSA) *S. aureus*.

The experimental design included four different steps: (i) preliminary determination of the minimum inhibitory concentration (MIC) of each compound; (ii) assessment of their antimicrobial activity in combination with an antibiotic; (iii) determination of their antibiofilm properties in terms of both inhibition of biofilm formation and disruption of preformed biofilms and (iv) investigation of their activity against VBNC *S. aureus* forms.

## 2. Results

### 2.1. Chemistry

**Scheme 1:**
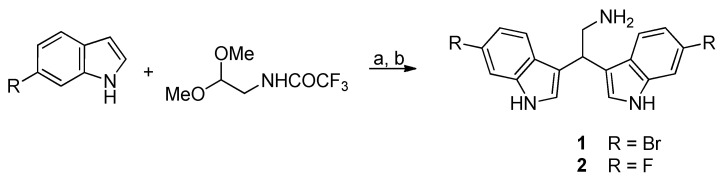
Reaction conditions: (**a**) diphenyl phosphate, acetonitrile, 80 °C, 24 h and (**b**) K_2_CO_3_, methanol, reflux, 2 h.

Marine bisindole alkaloid **1** and fluorinated analogue **2** were synthesized as previously reported [18] and described in Scheme 1.

### 2.2. Antibacterial and Adjuvants Activities of Bisindoles **1** and **2**

The MIC of the compounds **1** and **2** against the MRSA (*S. aureus* CH 10850) and the MSSA (*S. aureus* ATCC 29213) strains is reported in Table 1, along with their hemolytic activity. In detail, the MIC of compound **1** was 2 µg/mL for both *S. aureus* strains, while compound **2** exhibited MIC values of 32 and 16 µg/mL against *S. aureus* CH 10850 and *S. aureus* ATCC 29213, respectively. The toxicity of both compounds toward mammalian cells, assessed by determining their ability to lyse human erythrocytes, resulted to be very low, with values of 2.96% ± 0.02% and 2.32% ± 0.06%, respectively.

When tested in association with oxacillin against *S. aureus* CH 10850, compound **1** caused a MIC reduction from 256 to 128 μg/mL, with a ΣFICs = 1.0, indicating additivity (Table 2). More promising results were obtained with the combination oxacillin-compound **2**, that showed a MIC decrease of oxacillin (from 256 to 1 μg/mL) with ΣFICs of 0.5, indicating a synergistic effect (Table 2).

### 2.3. Antibacterial Activity in Plasma

**Figure 2:**
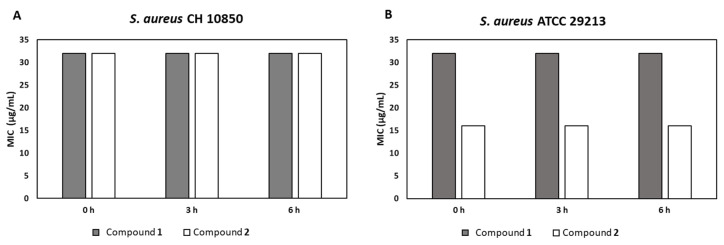
MIC of compounds **1** and **2** against *S. aureus* CH 10850 (**A**) and *S. aureus* ATCC 29213 (**B**), after preincubation in 50% blood plasma for 0, 3 and 6 h at 37 °C. Conservative estimates of three trials for each compound are shown.

One of the main issues with indole and their derivatives is their instability in vivo due to oxidation [22,23]. Thus, the MIC of both compounds was evaluated after preincubation (0, 3 and 6 h) in 50% human plasma at 37 °C. MIC values of compound **2** were in line with what found in both *S. aureus* strains, while a 16-fold increase was observed for compound **1** (Figure 2). However, it is very remarkable that all antimicrobial activities remained constant even after 6 h of plasma preincubation, hence showing a good stability in the physiologically relevant time intervals (Figure 2).

### 2.4. Antibiofilm Activity

Compound **1** was able to inhibit the biofilm formation of *S. aureus* CH 10850 and *S. aureus* ATCC 29213 at their MIC values (2 μg/mL), causing a biofilm reduction of 52.6% and 49.6%, respectively (Table 3). When tested at 2× MIC (4 μg/mL), no biofilm formation was observed in the wells after 24 h of incubation (100% of biofilm formation inhibition). Compound **2** at its MIC (32 μg/mL) was able to inhibit (61.0%) the biofilm formation of *S. aureus* CH 10850, reaching a 76.5% inhibition when used at 64 μg/mL (2× MIC). *S. aureus* ATCC 29213 biofilm formation, although less susceptible (58.9% inhibition) than *S. aureus* CH 10850 to the MIC (16 μg/mL) of compound **2**, was completely (100%) inhibited by 32 μg/mL (2× MIC) of the same compound (Table 3). Concerning the biofilm-disrupting ability, compound **1** successfully removed 37.5% of *S. aureus* CH 10850 biofilm and the 28.0% of *S. aureus* ATCC 29213 biofilm, after a 30 min treatment. Under the same experimental conditions, compound **2** exerted a greater disaggregating activity, removing 56.3% and 53.9% of preformed biofilm of *S. aureus* CH 10850 and *S. aureus* ATCC 29213 biofilms, respectively (Table 3).

### 2.5. VBNC Forms Induction

**Figure 3:**
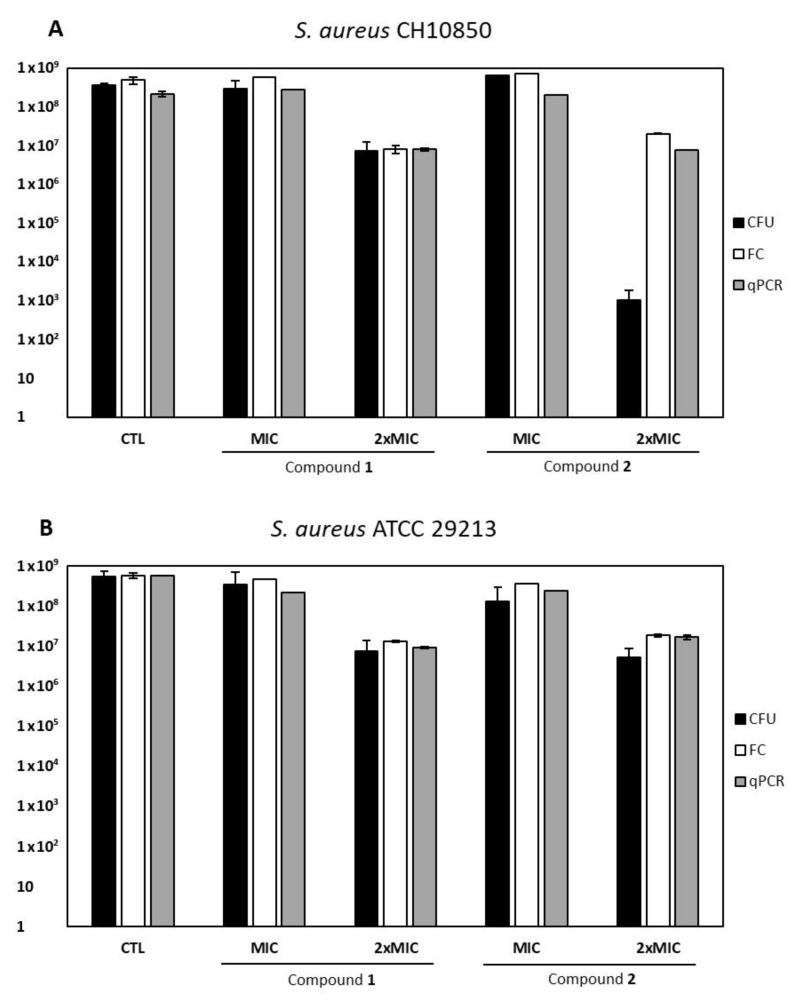
Effects of compounds **1** and **2** on *S. aureus* cell populations in in vitro biofilms. Biofilms of both *S. aureus* CH10850 (**A**) and *S. aureus* ATCC 29213 (**B**), in the absence (CTL) or presence of the two compounds at concentrations 1× and 2× MIC were developed in vitro and the amount of total viable and culturable cells counted after 24 h incubation at 37 °C. The amount of culturable cells was determined by plate count (CFU) and that of total (i.e., culturable and non-culturable) viable cells by qPCR and/or flow cytometry (FC).

The influence of compounds **1** and **2** on VBNC *S. aureus* cells induction was tested. Both strains exhibited an about 2log reduction of viable cells when exposed to 2× MIC of compound **2** (Figure 3). However only *S. aureus* 10850 showed a significant gap (corresponding to the VBNC amount) between total viable and culturable cells. Compound **1** did not seem to induce VBNC forms, as demonstrated by the lack of discrepancy between culture and qPCR/flow cytometry (Figure 3).

## 3. Discussion

Considering the wide spread of antimicrobial resistance, the search for new and more efficient therapeutic approaches against MRSA strains represents a priority. Several studies have evidenced that molecules based on indole scaffolds can be used against different bacterial species [18,24,25]. Recently, it has been demonstrated that selected marine-alkaloid-derived molecules possess adjuvant activity against multi drug resistant bacteria [19], thus opening new challenges in this field of investigation. Therefore, we tested the fluorinated bisindole **2**, as well as the lead natural compound **1**, toward two different—one susceptible and one resistant to methicillin—strains of *S. aureus* (MSSA ATCC 39213 and MRSA CH 10850, respectively). Both compounds showed an antimicrobial activity, although compound **1** resulted in being more efficient than its fluorinated analogue compound **2** (MIC, 2 vs. 32 µg/mL in the MRSA and MIC 2 vs. 16 µg/mL in the MSSA). Indeed, bisindoles are known to act as antimicrobials by two possible mechanisms of action: the positive charge on the nitrogen at physiological pH makes them cationic surfactants able to destabilize the cytoplasmic membrane. Moreover, they could possibly inhibit the bacterial pyruvate kinase as reported for similar bisindoles [26,27].

Our compounds were tested as possible adjuvants. Both restored oxacillin activity against the MRSA strain. Indeed, while tested in association with oxacillin, an additive effect (ΣFICs = 1.0) and a synergistic one (ΣFICs = 0.5), for compounds **1** and **2**, respectively, was found. Interestingly, the fluorine substitution in compound **2** was able to increase the adjuvant property of the natural compound. In detail, the fluorinated bisindole **2** was able to decrease the oxacillin MIC by 256-fold (from 256 to 1 µg/mL) for the clinical MRSA strain studied here. To the best of our knowledge, an adjuvant property of this class of bisindoles has never been reported, hence, the data herein observed provide novel information and stress the potential use of these class of natural compounds, together with their synthetic analogues, to contrast antibiotic resistance. Based on these encouraging results, the toxicological aspect of both compounds was also investigated by assessing their ability to lyse human erythrocytes. Neither of the two compounds exhibited hemolytic activity, thus adding important information on their safety. Although a different behavior after plasma incubation was observed (i.e., 16-fold MIC increase of compound **1**), it is of note that both compounds retained their antimicrobial activities even after 6 h of preincubation in blood plasma, demonstrating their chemical stability to this body fluid at physiologically relevant time intervals.

Microbial biofilms generate serious human health problems, including infectious diseases such as endocarditis, periodontitis and bacteremia [4]. The obtained results evidenced the ability of the tested compounds to inhibit *S. aureus* biofilm formation, reaching in most cases the complete biofilm formation inhibition at 2× MIC concentration, as previously reported for similar compounds [18]. Only for *S. aureus* CH 10850 a lower biofilm formation inhibition was evidenced, stressing the higher resistance of MRSA to antimicrobials [4]. Considering the important role of indole in bacteria, the presence of two units of indole in both compounds **1** and **2** may suggest that the observed antibiofilm activity derives from a modulation of indole-based signaling pathways [18,28]. Indeed, it was found that intracellular indole and its derivatives can cause a temporary repression of the *agr*-quorum sensing system in *S. aureus* [29]. The eradication activity of compounds **1** and **2** was afterward assessed on preformed biofilms of *S. aureus* CH 10850 MRSA and *S. aureus* ATCC 39213. As shown, the fluorinated bisindole **2** had a most pronounced disaggregating activity (>50% for both the examined strains) compared to the natural compound **1** (maximum 37.5%). This result could be related to the presence of two fluorine atoms in compound **2**, conferring a reduced lipophilicity compared to the two bromine atoms of the natural product **1**. To possibly confirm this hypothesis, we calculated an important physicochemical property related to the lipophilicity/hydrophilicity of a molecule, the octanol–water portion coefficient (logP). LogP values are high for lipophilic molecules and low for hydrophilic ones. Calculated LogP (cLogP) were 2.36 for compound **2** and 3.68 for compound **1** (by OSIRIS Property Explorer) [30]. From the cLogP values, fluorinated derivative **2** is more than 10-fold less lipophilic than the natural product **1**, and therefore more soluble in the hydrophilic biofilm polysaccharide matrix, resulting in a more effective disaggregating activity on preformed biofilms.

Biofilm development can also lead to the induction of dormant cells, persistent bacterial phenotypes that seem to be suitable adjuvant targets [11]. Between the two tested molecules, compound **2** exerted a VBNC inducing effect at a 2× MIC concentration. Moreover, the bacterial response resulted strain-specific, as *S. aureus* ATCC 29213 exhibited a little difference between total viable and culturable cells (0.5 log), whereas *S. aureus* CH 10850 (MRSA) showed a four-log difference between qPCR/flow cytometry and CFU counts. This seems to indicate a more powerful action of compound **2** against this specific strain, as already suggested by the checkerboard assay, resulting in synergy. Indeed, this compound caused the same reduction of total viable cells in both strains, but CH 10850 exhibited a deeper state of dormancy (i.e., higher amount of VBNC forms) than the ATCC 29213 strain. These data confirm the role of methicillin resistance in the development of VBNC forms as a survival strategy to stress conditions [31]. Furthermore, the expression of the *mecA* gene, conferring methicillin resistance through PBP2a synthesis, has been suggested to correlate with a greater stability of VBNC forms of the same strain (*S. aureus* CH10850) under unfavorable conditions [32]. It is thus conceivable that compound **2** may exert antibiofilm activity even if it can constitute a stress factor able to induce VBNC *S. aureus* forms, as previously described for different antimicrobial compounds [31].

To briefly summarize, natural bisindole alkaloid **1** and its fluorinated derivative **2** showed antimicrobial activity against the tested *S. aureus* strains (MIC ranging from 2 to 32 µg/mL). Surprisingly, compound **2** (at 16 µg/mL) reduced the MIC of oxacillin from 256 to 1 µg/mL (ΣFICs = 0.5) for the clinical MRSA strain. Although both molecules inhibited biofilm formation of *S. aureus* strains, compound **2** showed greater eradicating activity on preformed biofilm compared to the natural alkaloid **1**. None of the tested molecules exerted a VBNC inducing effect at their MIC values. Moreover, both compounds exhibited no hemolytic activity and a good stability in plasma, indicating a non-toxic profile. Although the tested compounds as well as the number of bacterial strains were limited, these preliminary results encourage us to further examine this class of interesting alkaloids, and in particular the fluorinated bisindole **2**, for in vivo applications to restore antibiotic treatment against MRSA infections.

## 4. Materials and Methods

### 4.1. Chemistry

All organic solvents used in this study were purchased from Sigma–Aldrich (St. Louis, MO, USA), Alfa Aesar (Haverhill, MA, USA), or TCI (Tokyo, Japan). Prior to use, acetonitrile was dried with molecular sieves with an effective pore diameter of 4 Å. Column chromatography purifications were performed under ‘‘flash” conditions using Merck (Darmstadt, Germany) 230–400 mesh silica gel. Analytical thin-layer chromatography (TLC) was carried out on Merck silica gel plates (silica gel 60 F254), which were visualized by exposure to ultraviolet light and an aqueous solution of cerium ammonium molybdate (CAM). ESI-MS spectra were recorded with a Waters (Milford, MA, USA) Micromass ZQ spectrometer. ^1^H NMR and ^13^C NMR spectra were recorded on a Bruker (Billerica, MA, USA) AC 400 or 100, respectively, spectrometer and analyzed using the TopSpin 1.3 (2013) software package. Chemical shifts were measured by using the central peak of the solvent.

#### 4.1.1. General Procedure for the Synthesis of Derivatives **1**–**2**

Diphenyl phosphate (0.02 mmol) was added to a solution of the appropriate indole derivative (0.4 mmol) and (trifluoroacetylamino)acetaldehyde dimethyl acetal (0.2 mmol) in anhydrous acetonitrile (0.2 mL), and the resulting mixture was stirred at 80 °C for 24 h in a sealed tube, monitoring the progress of the reaction by TLC and HPLC-MS. After cooling to room temperature, saturated aqueous NaHCO_3_ (30 mL) and dichloromethane (30 mL) were added and the two phases were then separated. The aqueous solution was extracted with dichloromethane (3 × 20 mL). After drying over dry Na_2_SO_4_, the combined organic phases were concentrated in vacuum and the resulting crude product was utilized without further purification. A mixture of that crude trifluoroacetamide derivative and potassium carbonate (1 mmoL) in MeOH (1.87 mL) and H_2_O (0.13 mL) was stirred and heated at reflux for 2 h. The MeOH was removed under reduced pressure and water was added (30 mL). The aqueous solution was extracted with dichloromethane (3 × 30 mL) and the resulting solution was dried with Na_2_SO_4_ and then concentrated in vacuum. The crude material was purified by flash chromatography on silica gel.

#### 4.1.2. 2,2-Bis(6-bromo-1*H*-indol-3-yl)ethanamine (**1**)

The physicochemical data of compound **1** are in agreement with those that were reported [18].

#### 4.1.3. 2,2-Bis(6-fluoro-1*H*-indol-3-yl)ethanamine (**2**)

Compound **2** was prepared employing 6-fluoro-1*H*-indole and was isolated by column chromatography (dichloromethane/methanol/ammonia, 95:4:1) as a white solid in 70% yield (two steps). TLC: Rf = 0.18 (silica gel; dichloromethane/methanol/triethylamine, 90:9:1; UV, CAM). MS (ESI): *m*/*z* 310 [M-H]^−^. ^1^H NMR (400 MHz, CD_3_OD, 293 K): δ = 3.37–3.41 (m, 2H, CHC*H_2_*NH_2_), 4.52 (dd, 1H, *J*_1_ = *J*_2_ = 7.5 Hz, C*H*CH_2_NH_2_), 6.72 (ddd, 2H, *J*_5−7_ = 2.0 Hz, *J*_5−4_ = 9.0 Hz, *J*_5−F_ = 9.5 Hz, H5), 7.04 (dd, 2H, *J*_7−5_ = 2.0 Hz, *J*_7−F_ = 9.5 Hz, H7), 7.14 (d, 2H, *J* = 3.0 Hz, H2), 7.44 (dd, 2H, *J*_4−F_ = 5.0 Hz, *J*_4−5_ = 9.0 Hz, H4) ppm. ^13^C NMR (100 MHz, CD_3_OD, 293 K): δ = 37.2, 45.6, 96.7 (d, 2C, *J* = 26 Hz, C5), 106.5 (d, 2C, *J* = 24 Hz, C7), 116.4 (2C, C3), 119.5 (d, 2C, *J* = 10 Hz, C4), 122.3 (d, 2C, *J* = 3 Hz, C9), 123.6 (2C, C2), 137.0 (d, 2C, *J* = 12 Hz, C8), 159.7 (d, 2C, *J* = 233 Hz, C6) ppm. The main physicochemical data of compound **2** are in agreement with those published [18].

### 4.2. Bacterial Strains

*S. aureus* CH 10850 (MRSA) [33] and *S. aureus* ATCC 29213 (MSSA), belonging to the strain collection of the Department of Life and Environmental Sciences (DiSVA), Polytechnic University of Marche (Ancona, Italy), were used. All the strains were cultured in brain heart infusion (BHI) broth or agar (Oxoid, Basingstoke, UK), subcultured in mannitol salt agar (MSA; Oxoid) and stored at −80 °C in BHI broth supplemented with 20% glycerol.

### 4.3. Determination of Minimum Inhibitory Concentration (MIC)

The minimum inhibitory concentration (MIC) of each molecule was determined by the microdilution method [34], with minor modifications. Bacteria were grown for 6 h in BHI broth at 37 °C, then diluted in Mueller Hinton II (Oxoid) to obtain ca. 5 × 10^5^ CFU/mL in 100 μL, in the presence of increasing concentrations (2–128 µg/mL) of each compound dissolved in molecular biology grade dimethyl sulfoxide (DMSO, Sigma). Positive and negative controls included MHB inoculated or not with bacterial suspensions, respectively. Preliminary assays were performed to exclude the possible bacteriostatic and/or bactericidal activity of the solvent (i.e., DMSO); in any case, the volume of DMSO never exceeded 5% (*v/v*) of the final total volume. Tetracycline was used as a reference antibiotic, for comparison. MIC was defined as the lowest concentration of compound able to inhibit bacterial growth after 24 h of incubation at 37 °C, as detected by the unaided eye. All the experiments were performed three times using independent cultures.

### 4.4. Checkerboard Assays

The synergy of the two compounds and oxacillin (Sigma–Aldrich, St. Louis, Missouri, USA) against MRSA *S. aureus* CH 10850 was evaluated by the checkerboard assays [35], performed using 2-fold increasing concentrations of both compound (from 8 to 0.125µg/mL for compound **1** and from 512 µg/mL to 8 µg/mL for compound **2**) and oxacillin (from 512 to 0.5 µg/mL). Since the two compounds were resuspended in DMSO, the upper limit of the concentrations range tested was determined considering a final concentration of 1% DMSO. The combinations of each compound and oxacillin were evaluated by fractional inhibitory concentration (FIC) index, interpreted as follows: ≤0.5, synergy; > 0.5 and ≤ 1.0, additive; >1.0 and <4, indifferent and ≥4, antagonistic.

### 4.5. Hemolytic Activity

The hemolytic activity of both compounds was evaluated as described by Ghosh et al. [36]. Briefly, 4 mL of freshly drawn, heparinized human blood was diluted with 25 mL of phosphate buffered saline (PBS), pH 7.4. After washing three times in 25 mL of PBS, the pellet was resuspended in PBS to 20 vol %. A 100 μL amount of erythrocyte suspension was added to 100 μL of different concentrations of compounds **1** and **2,** respectively. PBS and 0.2% Triton X-100 were used as the negative and positive control, respectively. Each condition was tested in triplicate. After 1 h of incubation at 37 °C each well was centrifuged at 1200 × *g* for 15 min, the supernatant was diluted 1:3 in PBS and transferred to a new plate. The OD_350_ was determined using the Synergy HT microplate reader spectrophotometer (BioTek, Winooski, VT, USA). The hemolysis (%) was determined as follows:

[(*A* − *A*_0_)/(*A*_total_ − *A*_0_)] × 100 (1)

where *A* is the absorbance of the test well, *A*_0_ the absorbance of the negative control, and *A_total_* the absorbance of the positive control; the mean value of three replicates was recorded.

### 4.6. Plasma Stability Assay

*S. aureus* CH 10850 and *S. aureus* ATCC 29213 were grown for 6 h in BHI broth and diluted in Mueller Hinton II to obtain a final concentration of 1.5 × 10^6^ CFU/mL. Fresh human blood was centrifuged at 3000 rpm for 5 min to separate the plasma from the cells. Three aliquots of compound **1** and compound **2** were dissolved in DMSO at a concentration of 128 and 1024 μg/mL, respectively and diluted 2-fold in plasma to reach the final concentration of 64 and 512 μg/mL. After incubation at 37 °C for 0, 3 and 6 h [36], 50 μL of each compound serially diluted 1:2 in MHB were added to a 96-well plate containing 50 μL of bacterial suspensions in MHB and incubated at 37 °C for 24 h. MIC values were determined as mentioned above [34]. No change in MIC values among the trials performed after different plasma-preincubation times was considered a proof of plasma-stability.

### 4.7. Biofilm Formation Inhibition

Biofilms were developed in 24-well polystyrene plates (VWR). *S. aureus* strains were grown in tryptic soy broth (TSB, VWR, Radnor, PA, USA) at 37 °C for 24 h. The bacterial concentration was adjusted to 5 × 10^6^ CFU/mL, as previously described, and 100 µL of each bacterial suspension were inoculated in 24-well polystyrene plates supplemented with the corresponding amount of the selected compounds at their MIC and 2× MIC values. Two wells were inoculated with bacteria in TSB, as controls. After 24 h of incubation at 37 °C, the wells were washed with PBS to eliminate unattached cells and covered with 0.1% (*v/v*) crystal violet (CV) dissolved in H_2_O for 15 min and then washed in PBS and air-dried. The remaining CV was dissolved in 85% ethanol for 15 min at room temperature and 200 µL from each well was transferred to a 96-well plate for spectrophotometric quantification at 570 nm (Multiscan Ex Microplate Reader, Thermo Scientific, Waltham, MA, USA). Each data point was averaged from at least 8 replicate wells. All assays were performed in triplicate using independent cultures.

### 4.8. Biofilm-Disrupting Activity

Biofilms of each *S. aureus* strain were prepared with the procedure described above. After 24 h of incubation at 37 °C, the biofilms were gently washed in PBS, covered with the right amount of each compound at its MIC value, and left in contact for 30 min. For each plate, two wells were treated with saline and used as negative controls. After treatment, the biomass was evaluated by CV staining as described above. All data were expressed as the mean of three independent experiments performed in duplicate.

### 4.9. VBNC Detection

To evaluate the induction of staphylococcal VBNC forms, *S. aureus* CH 10850 and *S. aureus* ATCC 29213 biofilms were developed in Petri dishes (Ø 35 mm) by inoculating OD_650_ = 0.1 cultures in BHI broth, alone or supplemented with either compound **1** or compound **2** at their MIC and 2× MIC concentrations, and incubated at 37 °C for 24 h. At the end of the incubation, biofilms were gently washed with 1 mL of PBS to remove planktonic bacteria, detached and then resuspended in 1 mL of PBS.

#### 4.9.1. Culture-Based Detection of Staphylococci

To evaluate the amount of the culturable cells, ten-fold serial dilutions of each mechanically detached biofilm were performed. For all dilutions 100 μL were spread onto BHI agar plates, incubated at 37 °C for 24 h prior to the enumeration of CFU.

#### 4.9.2. Flow Cytometry Detection of Staphylococci

The abundance of total viable staphylococci, both culturable and non-culturable, was determined by flow cytometry. Assays were performed using 200 µL of a 1:1000 dilution of detached *S. aureus* biofilms after live/dead staining (1× SYBR Green and 40 µg/mL propidium iodide), in a Guava Millipore cytometer, and analyzed by the GUAVASOFT 2.2.3 software. To discriminate bacterial cells from the background, a gate for cell detection in side scatter and green fluorescence (GRN) was applied, using both channels at 488 nm and a threshold value in the GRN channel; SYBR green and propidium iodide fluorescence were excited using a 488 nm laser and collected at 525/30 and 617/30 nm, respectively. To better detect signals, they were logarithmically (4 decades) amplified and, to increase statistical significance, the total number of particles analyzed was set to 20,000 events/replicate. All assays were run in duplicate.

#### 4.9.3. qPCR Detection of Staphylococci

Total DNA was extracted from 1 mL of biofilm aliquots diluted 1:10 (0× and 1× MIC) or undiluted (2× MIC) in PBS. Aliquots were centrifuged at 16,000 × *g* for 7 min, resuspended in 1 mL of STE (Tris-HCl 10 mM, NaCl 100 mM EDTA 1 mM) buffer supplemented with sucrose 20%, lysozyme 2.5 mg/mL and lysostaphin 100 µg/mL, and incubated for 1 h at 37 °C. Then, each aliquot was centrifuged, resuspended in 100 µL of PBS and the DNA was extracted using the QiaAmp DNA kit (Qiagen, Venlo, The Netherlands) according to the manufacturer instructions; a final elution volume of 80 µL was used.

*S. aureus* abundance was determined by *nuc*-qPCR using a Qiagen’s Rotor-GeneQ MDx thermocycler, 0.2 μM of each primer [31] 10 μL of 2 × Rotor-Gene SYBR Green PCR master mix (Qiagen), and 2 μL DNA. Cycling conditions were 95 °C for 5 min, followed by 35 cycles of 95 °C for 10 s, 60 °C for 10 s and 72 °C for 10 s. A melting curve was obtained by ramping the temperature from 59 to 95 °C (0.5 °C/10 s) and analyzed with Qiagen’s Rotor-GeneQ MDx software. DNA of *S. aureus* CH10850 and RNase-free water were used as positive and negative controls, respectively.

The number of viable *S. aureus* cells was determined as previously described [37,38]. Considering that *nuc* is a single copy gene [39] the amount of amplified DNA (ng) was divided for the weight (2.38928 × 10^−10^ ng) of *nuc*; and divided by 2 (qPCR template volume) to obtain the number of *S. aureus* cells corresponding to 1 µL of DNA extract. Staphylococcal abundance/mL of the original sample was then calculated multiplying the number of bacterial cell/µL of DNA extract by 80 (undiluted samples) or 800 (1:10 diluted samples), respectively. Plate counts were compared with both qPCR and flow cytometry quantifications; any discrepancy > 0.5 log was considered to attest the presence of a VBNC *S. aureus* subpopulation.

## 5. Conclusions

Marine alkaloid **1** and its fluorinated derivative **2** showed antimicrobial activity and inhibited biofilm formation of *S. aureus* strains. Moreover, bisindole **2** showed greater disaggregating activity (up to 56% for the examined strains) on preformed biofilm compared to the natural alkaloid **1**. Interestingly, compound **2** enabled 256-fold reduction in the MIC of oxacillin for the clinical MRSA strain herein studied. These encouraging data for analogue **2**, together with the evidences of its safety and stability, could represent the first step toward validating the potential of employing this adjuvant to restore oxacillin efficacy against MRSA infections.

## Figures and Tables

**Table 1:** Antimicrobial activity (minimum inhibitory concentration (MIC), µg/mL) and hemolytic activity (%) of the examined compounds **1** and **2**.

Compound	MIC (µg/mL)		Hemolysis (%)
	*S. aureus* CH 10850 (MRSA)	*S. aureus* ATCC 29213	
**1**	2	2	2.96 ± 0.02
**2**	32	16	2.32 ± 0.06

**Table 2:** Reduction of oxacillin resistance in *S. aureus* CH 10850 (methicillin resistant *Staphylococcus aureus* (MRSA)) exerted by compound **1** and **2** and determination of the synergistic activity as assessed by checkerboard assay (ΣFICs).

Compound	Concentration (µg/mL)	Oxacillin MIC (µg/mL)	ΣFIC	
None	-	256		
**1**	1	128	1.0	Additive
**2**	16	1	0.5	Synergistic

**Table 3:** Antibiofilm activities of compounds **1** and **2** against *S. aureus* CH 10850 and *S. aureus* ATCC 29213.

*S. aureus* Strain	Biofilm Formation Inhibition				Disaggregating Ability	
	Compound 1		Compound 2		Compound 1	Compound 2
	MIC	2× MIC	MIC	2× MIC	MIC	MIC
CH 10850 (MRSA)	52.6%	100%	61.0%	76.5%	37.5%	56.3%
ATCC 29213	49.6%	100%	58.9%	100%	28.0%	53.9%
